# Effect of mandibular mobilization on electromyographic signals in muscles of mastication and static balance in individuals with temporomandibular disorder: study protocol for a randomized controlled trial

**DOI:** 10.1186/1745-6215-14-316

**Published:** 2013-10-01

**Authors:** Yasmin El Hage, Fabiano Politti, Dowglas F Magalhães de Sousa, Carolina Marciela Herpich, Igor Phillip dos Santos Gloria, Cid André Fidelis de Paula Gomes, Ana Paula Amaral, Nívea Cristina de Melo, Thais Correa da Silva, Eric Edmur Camargo Arruda, Cesar Ferreira Amorim, Inaê Caroline Gadotti, Tabajara Oliveira Gonzalez, Fausto Berzin, Sandra Kalil Bussadori, Marília Barbosa Santos Garcia, Bruno Roberto Borges Barbosa, Daniela Aparecida Biasotto-Gonzalez

**Affiliations:** 1 Postgraduate Program in Rehabilitation Sciences, Laboratório de Biodinâmica do Movimento Humano, Universidade Nove de Julho (UNINOVE), Av. Dr. Adolfo Pinto, 109 Água Branca, São Paulo, SP 05001-100, Brazil; 2 Department of Physical Therapy, Universidade Nove de Julho (UNINOVE), Av. Dr. Adolfo Pinto, 109. Água Branca, São Paulo, SP 05001-100, Brazil; 3 Master’s Program in Physical Therapy, Universidade da Cidade de São Paulo (UNICID), Rua Cesáreo Galeno, 475. Tatuapé, São Paulo, SP 03071-000, Brazil; 4 Department of Physical Therapy, Florida International University (FIU), AHC3-427, 11200S.W. 8th Street, Miami, FL 33199, USA; 5 College of Dentistry, State University of Campinas, Morphology, Av. Limeira, 901 Caixa Postal 52, Piracicaba, SP, Brazil

**Keywords:** Temporomandibular joint disorders, Electromyography, Postural balance, Complementary therapy, Physical therapy modalities

## Abstract

**Background:**

The stomatognathic system and dysfunction in this system may be related to postural control. The proposal of the present study is to assess the effect of mandibular mobilization in individuals with temporomandibular disorder using surface electromyography of the muscles of mastication and stabilometric variables.

**Methods/Design:**

A randomized, controlled, blind, clinical trial will be carried out, with the participants divided into three groups: 1) facial massage therapy (control group), 2) nonspecific mandibular mobilization and 3) specific mandibular mobilization. All groups will be assessed before and after treatment using the Research Diagnostic Criteria for Temporomandibular Disorders, surface electromyography of the masseter and temporal muscles and stabilometry. This study is registered with the Brazilian Registry of Clinical Trials (RBR9x8ssz).

**Discussion:**

A large number of studies have employed surface electromyography to investigate the function/dysfunction of the muscles of mastication and associations with signs and symptoms of temporomandibular disorders. However, it has not yet been determined whether stabilometric variables offer adequate reliability in patients with this disorder. The results of the proposed study will help determine whether specific and/or nonspecific mandibular mobilization exerts an effect on the muscles of mastication and postural control. Moreover, if an effect is detected, the methodology defined in the proposed study will allow identifying whether the effect is local (found only in the muscles of mastication), global (found only in postural control) or generalized.

## Background

Correlations between dental occlusion and bodily balance have recently been analyzed [1-4]. The possibility of an association between conditions affecting the stomatognathic system, such as temporomandibular disorder (TMD), and postural problems have also been investigated [1,5-8]. Deviations in global posture lead to bodily adaptations and realignments, which can affect the organization and function of the temporomandibular joint (TMJ) [9]. Moreover, the effect of alterations in the muscles of mastication and dentoalveolar ligaments on the stomatognathic system can lead to a perturbation of visual stabilization, generating postural imbalance [2].

Constant oscillations (body sway) occur to maintain balance. Afferent (sensory) signals originate from proprioceptive, tactile, vestibular and visual receptors. Proprioception of the mandibular system emerges from the muscles of mastication (due to changes in the mandible position), receptors of the TMJ (due to rotation and/or translation of the condyles) and dentoalveolar ligaments (due to changes in occlusal contacts) and is determined by the trigeminal nerve [1,2,10-13].

Contact between the jaws is maintained by ligaments and muscles, including the masseter and temporal muscles. When these muscles are in tonic equilibrium, the mandible is at rest [2,14]. This mandibular resting position can be altered by different circumstances, such as occlusal interferences, TMD, the position of the head and body and emotional tension [14]. Electromyography (EMG) has evidenced a relationship between the stomatognathic system and cervical spine and how head movements [15], changes in body posture [14], occlusal contact pattern and the vertical dimension of the teeth [15-18] can alter EMG activity in the muscles of mastication and affect the mandibular resting position. Indeed, EMG activity in the muscles of mastication at rest in individuals with TMD may be greater than that found in individuals without this disorder, indicating muscle hyperactivity [19-21].

Studies have suggested that massaging the muscles of mastication, joint mobilization, manual therapy, mandibular exercises, biofeedback and the application of electrophysical resources may be effective in the treatment of TMD [22-26]. Joint mobilization has proven effective at reducing pain, disability and local ischemia [27], breaking fibrous adhesions, enhancing the extensibility of non-contractile structures, increasing the range of motion, enhancing the transmission of afferent information through the stimulation of mechanoreceptors and stimulating proprioception and the production of synovial fluid [27,28]. The use of joint mobilization has been tested on individuals with TMD, in whom a reduction in pain and increased mandibular range of movement (ROM) have been reported [29-32].

Massage has proven effective at diminishing EMG activity and pain [19,21], preventing the formation of connective tissue adherences, enhancing the production and circulation of endogenous opioids [19], increasing local blood flow, facilitating the elimination of residual substances and extracellular fluid, improving the nutrition of myofibrils and promoting relaxation and a sensation of wellbeing [33,34]. Massaging the muscles of mastication has been administered to patients with TMD, leading to a reduction in pain, an increase in mandibular ROM and a decrease in EMG activity in the muscles [21,35].

Postural control may be related to afferent signals of the trigeminal nerve, which depend on information from receptors of the stomatognathic complex (dentoalveolar ligaments, muscle and joint receptors). Moreover, TMD may alter muscle activity and the positioning of structures of the stomatognathic system, interfering in the information transmitted for the control of posture. Thus, relaxation of the muscles of mastication (as assessed through surface EMG) and the alignment of joint structures (assessed through a clinical exam) may alter afference from the receptors of the stomatognathic system, thereby affecting postural control (assessed through stabilometry). Based on this hypothesis, the aim of the proposed study is to determine the effect of facial massage and mandibular mobilization on EMG activity of the muscles of mastication and static balance in individuals with TMD.

## Methods/Design

### Overview of research design

A randomized, controlled, blind, clinical trial is proposed to study the effects of specific and nonspecific mandibular mobilization. The participants will be allocated to three groups through a randomization process using opaque envelopes. Each group will receive a specific form of treatment for TMD. The control group (CG) will receive massage therapy and those submitted to joint mobilization will be divided into two groups: the specific mobilization group (SMG) and the nonspecific mobilization group (NMG). Evaluations will be carried out before and after treatment using the Research Diagnostic Criteria for Temporomandibular Disorders (RDC/TMD), EMG of the masseter and temporal muscles and stabilometry.

After screening, individuals with TMD (based on the RDC/TMD) will be selected. The study will be divided into three types of treatment, three evaluations and two phases, to which all individuals will be submitted:

Evaluation 1: randomization of treatment with allocation to treatment groups, stabilometric analysis and EMG analysis, with the administration of the protocol for the respective treatment.

Evaluation 2: second stabilometric and EMG evaluation immediately following respective treatment.

Treatment phase: treatment following respective protocol.

Evaluation 3: third stabilometric and EMG evaluation and second administration of the RDC/TMD questionnaire.

### Flowchart (Figure 1)

**Figure 1:**
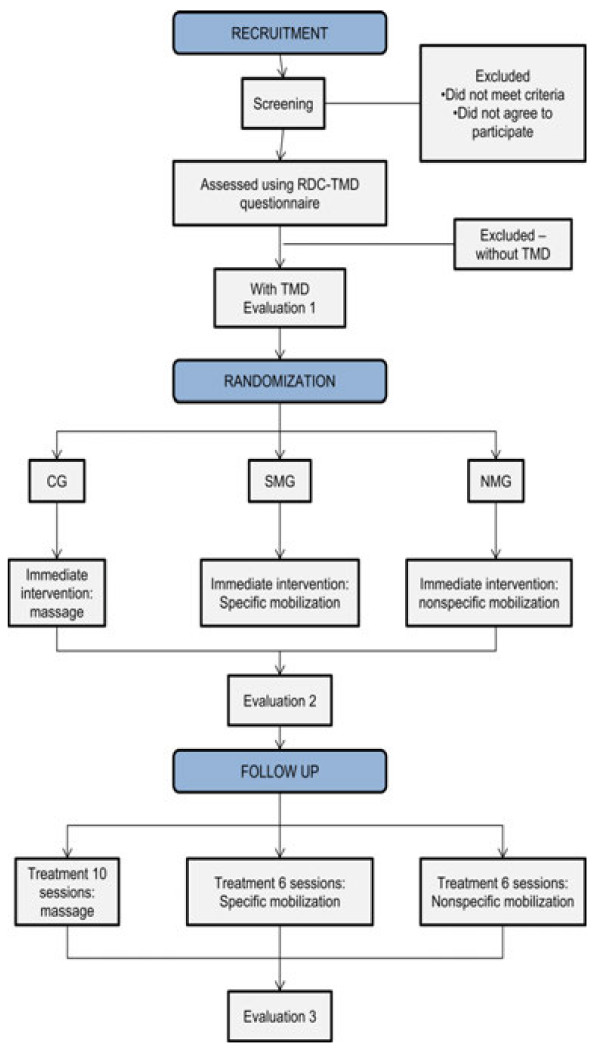
Method flowchart.

#### ***Blinding***

Participants will be blinded to the group allocation and will not be aware if they are being subjects of the treatment or control groups. The therapists will not be blinded because they will perform the interventional protocols. However, the therapists will not take part in the outcome measurements or the statistical analyses, and will be requested not to disclose details about their treatment to the outcome assessors or participants. The outcome assessors will be blinded to the randomization allocation and will not be involved in performance of the interventions. The statistician will be blinded to the group allocation until completion of the statistical analyses.

#### ***Inclusion criteria***

A diagnosis of TMD, complete dentition (except third molars) and mandibular deviation and/or deflection.

#### ***Exclusion criteria***

Crossbite, open bite, mandibular prognathism or retrognathism, denture use, current orthodontic or physiotherapeutic treatment, neurological disorder that may affect balance, use of orthopedic insoles.

### Ethical considerations

The proposed study received approval from the local Human Research Ethics Committee under process number 457625 dated 28 September 2011. The study will be conducted in compliance with the norms that regulate research involving human subjects contained in Resolution number 196/97 of the Brazilian National Health Council and is registered with the Brazilian Registry of Clinical Trials (RBR9x8ssz). All participants will be informed regarding the objectives and procedures of the study and will be asked to sign a statement of informed consent agreeing to participate.

### Procedures

#### ***Research diagnostic criteria for temporomandibular disorders - RDC/TMD***

The RDC/TMD is a biaxial diagnostic tool [36] composed of a clinical exam based on a detailed physical evaluation of the mouth opening pattern, vertical extension of mandibular movement, noises in the TMJ upon palpation during vertical movement, excursive mandibular movements and noises in the TMJ upon palpation during lateral excursion and protrusion. The clinical diagnosis is divided into three categories [37,38] (Table 1). The RDC/TMD questionnaire is made up of 31 items addressing general health, oral health, history of facial pain, mouth opening limitation, joint noises, habits, bite, ringing in the ears, health conditions in general, joint problems, headache, current behavior and social and economic profile.

**Table 1:** Classification and diagnosis of temporomandibular disorder (TMD) subgroups based on Research Diagnostic Criteria for Temporomandibular Disorder (RDC/TMD)

**Group**	**Subgroup**
I	A. myofascial pain
	B. myofascial pain with limited opening
	No group I diagnosis
II right	A. disk displacement with reduction
	B. disk displacement without reduction with limited opening
	C. disk displacement without reduction without limited opening
	No group II diagnosis
II left	A. disk displacement with reduction
	B. disk displacement without reduction with limited opening
	C. disk displacement without reduction without limited opening
	No group II diagnosis
III right	A. arthralgia
	B. osteoarthitis of TMJ
	C. osteoarthosis of TMJ
	No group III diagnosis
III left	A. arthralgia
	B. osteoarthitis of TMJ
	C. osteoarthosis of TMJ
	No group III diagnosis

#### ***Stabilometry***

The participants will remain barefoot on a force plate (Biomec 400 v1.1^®^, EMG System Ltda^®^ - http://www.emgsystem.com.br - Rua Porto Principe, 50 - Vila RubiCEP12245-572-São José dos Campos/SP. Phone: 55 12 3922-4069/55 12 3942–4736) consisting of four load cells with an internal circuit that changes in electrical resistance upon the application of force (dimensions: 1 m × 1 m; sampling frequency: 100 Hz). The participants will stand with unrestricted width of the foot base, heels in alignment, arms alongside the body and gaze fixed on a circular target (5 cm in diameter), at the height of the glabellum, attached to a pedestal at a distance of two meters.

The stabilometric data will be recorded during quiet standing for 70 seconds under two visual conditions: eyes open (EO) and eyes closed (EC). Data collection will be carried out under each condition, with a one-minute rest between trials, during which time with subjects will be allowed to sit.

#### ***Surface EMG***

Surface EMG signals from the right and left masseter and right and left anterior temporal bundle will be recorded with disposable surface electrodes (Ag/AgCl - Noraxon^®^ - http://noraxon.com - 15770 N. Greenway-Hayden Loop, #100Scottsdale, AZ 85260. Phone: 480-443-3413) attached to the belly of the muscle in the region with the greatest tonus after the volunteer performs moderate intercuspation (clenching of the teeth). The inter-electrode distance will be 20 mm from center to center. The sites for the electrodes will be cleaned with a cotton ball soaked in alcohol to diminish the impedance between the skin and electrodes [39]. A rectangular metallic electrode measuring 3 cm × 2 cm coated with Lectron II conductive gel (Pharmaceutical Innovations^®^ - http://www.pharminnovations.com - 897 Frelinghuysen Ave Newark, NJ 07114 (973) 242–2903) to increase the conduction capacity and impede interference from external noise will be attached to the left wrist of the volunteers for reference.

The bipolar EMG signals will be amplified using an eight-channel module (EMG System do Brasil Ltda^®^ - http://www.emgsystem.com.br - Rua Porto Principe, 50 - Vila RubiCEP12245-572-São José dos Campos/SP. Phone: 55 12 3922-4069/55 12 3942–4736)) consisting of a conditioner with a band pass filter with cut-off frequencies of 20 to 1000 Hz, an amplifier gain of 1,000 and a common mode rejection ratio > 120 dB. All data will be acquired and processed using a 16-bit analog to digital converter (EMG System do Brasil Ltda^®^ - http://www.emgsystem.com.br - Rua Porto Principe, 50 - Vila RubiCEP12245-572-São José dos Campos/SP. Phone: 55 12 3922-4069/55 12 3942–4736)) at a sampling frequency of 2 kHz.

For the recording of the EMG signals, the participants will remain seated in a chair with feet apart and hands resting on the lower limbs. To standardize the EMG potentials of the four muscles analyzed with tooth contact, two strips of Parafilm M^®^ (American National Can TM, Chicago, IL, USA) will be folded into five parts (3 mm in thickness) and positioned on the first and second mandibular molars (bilateral) of each subject [40]. Four seconds of maximum voluntary clenching (MVC) will be recorded three times, with a three-minute interval between readings.

After five minutes of rest, the recording of EMG activity will be performed three times successively under the following conditions: i) rest position: the subjects will be asked to relax and maintain the maxillary and mandibular teeth without contact; ii) maximal intercuspation (isometric): the subjects will be ask to clench the teeth as hard as possible and maintain the same level of contraction (tooth contact); and iii) chewing (isotonic): the subjects will be instructed to mildly and symmetrically bite down (chew) on the two strips of Parafilm M^®^ (positioned bilaterally) in time with a metronome adjusted to 60 beats per minute.

For the EMG signals recorded in rest position and during chewing, a two-minute interval will be allowed between readings. For maximal intercuspation, a five-minute interval between readings will be used. The data collection time will be 15 seconds for the rest position and chewing condition and 8 seconds for maximal intercuspation.

#### ***Massage and mobilization interventions***

The participants will be divided into three groups, each of which will be submitted to a different protocol. Ten sessions will be given for each treatment.

Protocol 1: massage therapy, described in Table 2; the procedure will last for 20 minutes and consist of synchronized sliding and kneading with medium pressure on the anterior temporal and masseter muscles bilaterally.

Protocol 2: nonspecific mandibular mobilization with the participant in dorsal decubitus on a cot, performed by a previously trained, experienced therapist wearing disposable gloves, who will position the fifth finger on the last molar, performing nonspecific mandibular mobilization, grade I [41], intermittently for one minute, with five repetitions. In the interval between repetitions, the participant will perform mouth opening 15 times with the tongue on the incisive papilla.

Protocol 3: specific mandibular mobilization with the participant in dorsal decubitus on a cot, performed by a previously trained, experienced therapist wearing disposable gloves, who will position the fifth finger on the last molar of the side being treated and ask the participant to lightly bite down on the finger, at which point the therapist will perform specific mandibular mobilization for the disk with reduction or without reduction:

For disk with reduction, pressure to be applied will be in the caudal direction and the oscillation will be cranial to caudal, grade I [41], intermittently for one minute, with five repetitions. In the interval between repetitions, the participant will perform mouth opening 15 times with the tongue on the incisive papilla;

For disk without reduction, pressure to be applied will also be in the caudal direction and the oscillation will be caudal to cranial, grade I [41], intermittently for one minute, with five repetitions. In the interval between repetitions, the participant will perform mouth opening 15 times with the tongue on the incisive papilla.

**Table 2:** Twenty-minute facial massage protocol performed bilaterally

	
Surface sliding	3 minutes
Circular movements in counter-clockwise direction	
Anterior bundle of temporal muscle	
Kneading	3 minutes
Kneading in cranial-caudal direction	
Upper fibers of masseter muscle	
Return of hands to initial position with continuous caudal-cranial sliding of suprahyoid musculature	
Surface sliding	1 minute
Circular movements in counter-clockwise direction	
Anterior bundle of temporal muscle	
Kneading	1 minute
Kneading in cranial-caudal direction	
Upper fibers of masseter muscle	
Return of hands to initial position with continuous caudal-cranial sliding of suprahyoid musculature	
Surface sliding	1 minute
Circular movements in counter-clockwise direction	
Anterior bundle of temporal muscle	
Kneading	1 minute
Kneading in cranial-caudal direction	
Upper fibers of masseter muscle	
Return of hands to initial position with continuous caudal-cranial sliding of suprahyoid musculature	
Deep sliding	3 minutes
Circular movements in counter-clockwise direction	
Anterior bundle of temporal muscle	
Kneading	3 minutes
Kneading in cranial-caudal direction	
Upper fibers of masseter muscle	
Return of hands to initial position with continuous caudal-cranial sliding of suprahyoid musculature	
Deep kneading	1 minute
Circular movements in counter-clockwise direction	
Anterior bundle of temporal muscle	
Kneading	1 minute
Kneading in cranial-caudal direction	
Upper fibers of masseter muscle	
Return of hands to initial position with continuous caudal-cranial sliding of suprahyoid musculature	
Deep sliding	1 minute
Circular movements in counter-clockwise direction	
Anterior bundle of temporal muscle	
Kneading	1 minute
Kneading in cranial-caudal direction	
Upper fibers of masseter muscle	
Return of hands to initial position with continuous caudal-cranial sliding of suprahyoid musculature	
Finalization with light touch	

In protocols 2 and 3, the side of the mandible to be mobilized will be previously defined through the diagnosis performed with the RDC/TMD; depending on the diagnosis, one or both sides of the mandible may be mobilized. The therapist will remain standing on the contralateral side to the mandibular mobilization, thereby avoiding any pressure on the mandible and exclusively performing millimeter mandibular movements.

### Pilot studies

The sample size and stabilometric variables to be used in the study were defined in two independent pilot studies, as described below:

a) Sample description and characterization: pilot study 1. The sample was defined based on a pilot study involving eight women (mean age: 23.16 ± 3.61 years) with TMD, as diagnosed using the RDC/TMD (described in the Procedures subsection). The good reliability and reproducibility of EMG signal in the evaluation of the muscles of mastication [42-45] was the main criterion for the choice measure to calculate the sample size in the proposed study.Thus, the sample size was calculated considering the largest standard deviation (SD) and root mean square (RMS) of the normalized amplitudes of the EMG signal of the right and left masseter and temporal muscles. The data were collected during maximum intercuspation for eight seconds before (T0) and after five sessions of nonspecific mobilization (T1). Considering a statistical power of 0.80, the right temporal muscle determined the largest sample (13 individuals (T0 = 1.19; T1 = 1.08; SD = 0.14)). The sample will be made up of male and female individuals, between 18 and 36 years of age, selected based on the eligibility criteria and diagnosed with TMD based on the RDC/TMD.

b) Stabilometric analysis: pilot study 2 after the definition of the sample size, a second pilot study was conducted to determine the stabilometric variables that exhibit the best within-day and between-day reproducibility. Fifteen female subjects with TMD, as diagnosed by the RDC/TMD (described in Procedures subsection), were recruited. The subjects had a mean age of 20.13 (± 6.19) years, mean height of 1.64 cm (± 0.06) and mean body weight of 61.08 Kg (± 9.72). Each participant completed two test sessions on two different days. All sessions were performed in the same laboratory environment by the same examiners, with a seven-day interval between sessions. Each session consisted of two successive trials of quiet upright stance, with a rest period of approximately 60 seconds between trials. The stabilometric data were recorded for 70 seconds under two visual conditions: eyes open (EO) and eyes closed (EC). For the analysis of the stabilometric variables, the initial 10 seconds were discarded to avoid initial transients and anticipation effects. Two collections were carried out under each visual condition, with a one-minute rest between collections (session 1). The order of the visual conditions (EO and EC) was randomly determined to control for the learning effect.

c) The justification for two collections under each visual condition is based on the fact that three trials were initially planned for each visual condition, but the first five individuals selected for this initial study reported discomfort and fatigue. Thus, new tests were carried out with the same participants with a one-day interval and the trials were reduced to two under each visual condition, with no report of any discomfort. The possibility of diminishing the time to less than 60 seconds was not considered due to the indication of using at least 90 seconds of data collection for each trial [46]. Moreover, it has been demonstrated that, under the eyes closed condition, reliability is lesser for short sampling and rises as the individual adapts [47]. Thus, the measurements of center of pressure (CoP) of these five individuals were not considered and only two trials under each visual condition were performed in this pilot study to maintain the integrity of the participants in the study.The tests were repeated after a one-week interval (session 2). All details regarding the equipment and analysis of the stabilometric data are described in the Procedures and Signal processing subsections, respectively.

### Signal processing

#### ***Stabilometric parameters***

Displacement from the center of pressure (CoP) in the anteroposterior (AP) and mediolateral (ML) directions will be used to analyze body sway, as in the preliminary study (pilot study 2). CoP data will be filtered with a Butterworth low-pass filter with a cut-off frequency of 10 Hz [46]. Postural sway will be quantified by means of two common scale-dependent variables: CoP sway area as an indicator for magnitude of CoP movements and CoP mean velocity and frequency as indicators of the efficiency of postural control. As an indicator for the regularity of CoP movements, a scale-independent variable denominated sample entropy (SaEn) will be used. Each parameter is briefly described below:

– Sway area (cm^2^) will be estimated by fitting an ellipse to the CoP data (AP versus ML) that encompasses 95% of the data; [48]

– Mean velocity (cm/s) of the CoP in both AP and ML directions will be calculated by taking the total distance traveled and dividing it by the time of the trial; [49]

– Frequency (Hz) of CoP displacement will be determined by the frequency at which 80% of the CoP spectral power is below. The 80% value was chosen based on the study by Baratto *et al*. (2002) [50], who suggest that this value is a better discriminator for CoP data than other spectral measurements;

– SaEn is a method for quantifying the regularity of a time series [51,52] and reflects the conditional probability that two sequences of 'm’ consecutive data points that are similar to each other will remain similar when one more consecutive point is included. Being 'similar’ means that the value of a specific measure of distance is less than 'r’. Therefore, SaEn is a function of m and r parameters [53]. The embedding dimension, m, and the tolerance distance, r, will be set to m = 3 and r = 30% of standard deviation of the data sequence [54]. To render an outcome for the scale-independent SaEn, the CoP data in both AP and ML directions will be normalized to unit variance by dividing the time-series in question by their respective standard deviation [55]. Values close to zero indicate greater regularity, while values closer to two indicate greater entropy in the signal being analyzed. In the present study, a decrease in sample entropy (that is, more regular sway fluctuations) will be interpreted as a decrease in the effectiveness of postural control.

#### ***Surface electromyographic data***

Five seconds of the signal will be used for the calculation of the root mean square (RMS) of the amplitude, with the two initial seconds and final one second of the eight-second reading discarded. For data recorded in the resting position and during maximal intercuspation, the RMS will be calculated using a 200 ms moving window.

The data recorded during chewing will be normalized by the peak EMG recorded from the same isotonic trial (dynamic mean method). The amplitude of the signal obtained in the resting position and during maximal intercuspation will be expressed as percentage of the average RMS recorded in the three readings during maximum voluntary contraction (MVC) (%MVC).

All stabilometric and EMG signals will be processed performing specific routines carried out in the Matlab program, version 7.1 (The MathWorks Inc., Natick, MA, USA).

#### ***Data analysis***

The Shapiro-Wilk test will be used to test the data with regard to Gaussian distribution. Data that demonstrate parametric distribution will be expressed as mean and standard deviation (SD) values. Data with nonparametric distribution will be expressed as median and inter-quartile interval. Either repeated-measure ANOVA or Friedman’s test will be used for the intra-group analysis and either one-way ANOVA or the Kruskal-Wallis test will be used for the inter-group analysis of data with parametric and non-parametric distribution, respectively.

#### ***Reliability***

Data on the CoP measures obtained in two sessions without any type of intervention (pilot study 2) are expressed as mean and SD. Some test results revealed non-normal distribution. Thus all data were log-transformed prior to analyses to negate the effects of heteroscedasticity [56]. The reliability of each CoP-based measure was quantified using intraclass correlation coefficients (ICC) [57], 95% confidence intervals (CI) and the standard error of the mean (SEM) [58]. For the purposes of this study, the ICC was interpreted using the following criteria: 0.00 to 0.39 (poor), 0.40 to 0.59 (fair), 0.60 to 0.74 (good) and 0.75 to 1.00 (excellent) [59]. The SEM was used to express reliability in absolute values, with a lower SEM denoting greater reliability of the measurement, whereas a high SEM indicates a high level of error and implies the non-reproducibility of the tested values.

All data were analyzed using the Statistical Package for the Social Sciences (SPSS - http://www-01.ibm.com/software/analytics/spss/ - IBM Corporation 1 New Orchard Road Armonk, New York 10504–1722 United States 914-499-1900 Version 17.

## Results

Fifteen individuals with TMD were analyzed to determine the reliability of the stabilometric variables. However, the data on one individual were not computed in the results of this preliminary study. This decision was based on the fact that the CoP measurements were up to 60% greater than the overall mean of the group and artifacts were also observed in the signal.

Table 3 displays the mean and SD values of the stabilometric measures in the two sessions (log values). Table 4 displays the ICC, SEM and 95% CI for the within-and between-day reliability of the CoP measurements of all participants. Mean velocity (AP and ML) was the most reliable measure, achieving excellent within-day and between-day ICC values.

**Table 3:** Mean and standard deviation of stabilometric measures recorded in two sessions with a seven-day interval

		**Session 1**		**Session 2**	
		**Test**	**Retest**	**Test**	**Retest**
Area(cm/s^2^)	EO	0.76 ± 0.33	0.63 ± 0.30	0.75 ± 0.34	0.64 ± 0.33
	EC	0.84 ± 0.39	0.78 ± 0.36	0.79 ± 0.41	0.97 ± 0.58
V_AP_(cm/s)	EO	0.61 ± 0.14	0.60 ± 0.13	0.57 ± 0.10	0.60 ± 0.13
	EC	0.66 ± 0.15	0.66 ± 0.13	0.66 ± 0.15	0.65 ± 0.13
V_ML_(cm/s)	EO	0.55 ± 0.13	0.54 ± 0.14	0.56 ± 0.14	0.57 ± 0.14
	EC	0.56 ± 0.14	0.56 ± 0.14	0.54 ± 0.15	0.54 ± 0.14
ƒ80_AP_(Hz)	EO	0.60 ± 0.07	0.59 ± 0.09	0.62 ± 0.07	0.62 ± 0.09
	EC	0.62 ± 0.07	0.61 ± 0.07	0.62 ± 0.08	0.59 ± 0.06
ƒ80_ML_(Hz)	EO	0.74 ± 0.31	0.79 ± 0.32	0.79 ± 0.28	0.76 ± 0.25
	EC	0.72 ± 0.20	0.74 ± 0.32	0.75 ± 0.26	0.68 ± 0.16
SaEn_AP_	EO	0.20 ± 0.06	0.22 ± 0.10	0.19 ± 0.05	0.21 ± 0.06
	EC	0.20 ± 0.05	0.21 ± 0.08	0.21 ± 0.08	0.18 ± 0.05
SaEn_ML_	EO	0.43 ± 0.23	0.47 ± 0.18	0.41 ± 0.20	0.46 ± 0.19
	EC	0.43 ± 0.23	0.45 ± 0.20	0.44 ± 0.20	0.39 ± 0.18

AP and ML: anteroposterior and mediolateral directions; Area: CoP sway in AP and ML directions; *EC*: eyes closed *EO*: eyes open; *ƒ80*: median frequency; *SaEn*: sample entropy; *V*: mean velocity.

**Table 4:** Within-day and between-day reliability of center of pressure (CoP) measurements

		**Session 1**		**Session 2**		**Session 1 and 2**	
		**ICC (95% IC)**	**SEM**	**ICC (95% IC)**	**SEM**	**ICC (95% IC)**	**SEM**
Area(cm/s^2^)	EO	0.51 (0.04 to 0.81)	0.21	0.72 (0.33 to 0.90)	0.17	0.52 (0.26 to 0.78)	0.22
	EC	0.81 (0.51 to 0.94)	0.25	0.44 (-0.06 to 0.77)	0.36	0.48 (0.21 to 0.74)	0.30
Vel_AP_(cm/s)	EO	0.96 (0.88 to 0.99)	0.03	0.92 (0.73 to 0.97)	0.03	0.79 (0.58 to 0.91)	0.06
	EC	0.95 (0.84 to 0.98)	0.03	0.83 (0.54 to 0.94)	0.06	0.76 (0.53 to 0.89)	0.07
Vel_ML_(cm/s)	EO	0.98 (0.93 to 0.99)	0.02	0.96 (0.87 to 0.98)	0.03	0.96 (0.91to 0.98)	0.03
	EC	0.94 (0.82 to 0.98)	0.04	0.92 (0.78 to 0.98)	0.04	0.82 (0.65 to 0.93)	0.06
ƒ80_AP_(Hz)	EO	0.78 (0.45 to 0.91)	0.04	0.83 (0.56 to 0.94)	0.03	0.76 (0.56 to 0.90)	0.04
	EC	0.71 (0.33 to 0.90)	0.04	0.52 (0.05 to 0.81)	0.05	0.66 (0.43 to 0.85)	0.04
ƒ80_ML_(Hz)	EO	0.85 (0.61 to 0.94)	0.12	0.80 (0.49 to 0.92)	0.12	0.70 (0.46 to 0.86)	0.16
	EC	0.75 0.38 to 0.90)	0.12	0.50 (0.02 to 0.79)	0.14	0.57 (0.31 to 0.80)	0.15
SaEn_AP_	EO	-0.04 (-0.60 to 0.50)	0.16	0.46 (-0.05 to 0.78)	0.10	0.20 (-0.03to 0.54)	0.13
	EC	0.53 (0.01 to 0.52)	0.10	0.30 (-0.21 to 0.70)	0.12	0.54 (0.27 to 0.78)	0.10
SaEn_ML_	EO	0.65 (0.21 to 0.87)	0.12	0.71 (0.34 to 0.89)	0.10	0.67 (0.44 to 0.86)	0.11
	EC	0.63 (0.17 to 0.86)	0.13	0.49 (-0.03 to 0.89)	0.13	0.50 (0.22 to 0.77)	0.14

AP and ML: anteroposterior and mediolateral directions; Area: CoP sway in AP and ML directions; *CI*: confidence interval; *ƒ80*: median frequency; *ICC*: intraclass correlation coefficient; *SaEn*: sample entropy; SEM: standard error of mean; *V*: mean velocity; *EC*: eyes closed; *EO*: eyes open.

## Discussion

The main objective of the proposed study will be to assess the effect of mandibular mobilization using surface EMG and stabilometric variables. Numerous studies have substantiated the reliability and reproducibility of surface EMG in the evaluation of the muscles of mastication [42-45], which was the main criterion for the choice of this measure to calculate the sample size for the proposed study, and especially to investigate the possible effects of the two techniques for the treatment of TMD.

Regarding CoP measurements, however, it is not yet possible to determine whether stabilometric variables offer adequate reproducibility in patients with TMD. The reliability of CoP variables has generally been analyzed in healthy adults and elderly individuals [60] or those with associated diseases [61,62], but no such studies are found involving patients with TMD. This led to the need to conduct the aforementioned pilot study to test the reproducibility of the data and determine the collection protocol for the stabilometric variables idealized in the project. The preliminary execution of this project also contributed to an adjustment of the data collection protocol regarding the number of trials per session based on the participants’ reports of discomfort, as described above.

Studies have demonstrated that the number of recorded tests exerts an effect on the reliability of CoP measurements used to assess postural control during quiet standing (static posture) [60-63]. Although the results of this study indicate that CoP-based measurements have quite diverse levels of reliability, mean velocity was the most reliable measure with regard to within-day and between-day readings, which is consistent with earlier reports [46,60,61,63,64]. Within-day reliability was generally better than between-day reliability. Moreover, the reliability of CoP measurements was also influenced by the visual condition (eyes open and eyes closed). The ICC was greater under the eyes open condition for mean velocity and median frequency (ƒ80) in the two directions (AP and ML). The visual influence on postural control demonstrates a specific characteristic among individuals with TMD, as higher overall reliability values have previously been demonstrated under the eyes closed condition compared to eyes open [49,65,66].

The weakest index regarding reliability was the SaEn, followed by sway area. The variation in ICC among poor, fair and good and the high SEM demonstrate that these variables should not be considered when investigating the possible effects of mandibular mobilization for the treatment of TMD.

The results of this pilot studied showed that mean velocity was the most reliable measure, which is consistent with earlier reports, and can be selected as a reliable postural sway measure. Moreover, the results of the proposed study will help determine whether specific and/or nonspecific mandibular mobilization exerts an effect on the muscles of mastication and postural control. Moreover, if an effect is detected, the methodology defined in the proposed study will allow identifying whether the effect is local (found only in the muscles of mastication), global (found only in postural control) or generalized.

## Trial status

This trial is currently recruiting patients.

## Abbreviations

Ag/AgCl: Silver/silver chloride; ANOVA: Analysis of variance; AP: Anteroposterior; CG: Control group; CI: Confidence interval; CoP: Center of pressure; COPAP: Anteroposterior center of pressure; COPML: Mediolateral center of pressure; EC: Eyes closed; EMG: Electromyography; EO: Eyes open; f80: Median frequency; ICC: Interclass correlation coefficient; ML: Mediolateral; MVC: Maximum voluntary contraction; NMG: Nonspecific mobilization group; RDC/TMD: Research Diagnostic Criteria for Temporomandibular Disorders; RMS: Root mean square; ROM: Range of movement; SaEn: Sample entropy; SD: Standard deviation; SEM: Standard error of mean; EMG: Electromyography; SPSS: Satistical package for social sciences; TMD: Temporomandibular disorder; TMJ: Temporomandibular joint.

## Competing interests

The authors declared that they have no competing interests.

## Authors’ contributions

YE submitted the manuscript. YE, FP, DABG, TO and CAFPG drafted the original protocol. TO, FP, ICG and DABG contributed to the study design. FP, DABG and YE performed the sample size calculation and description of the data processing. DFMS, SKB, FB - dentists in charge of screening and application of diagnostic criteria; CAFPG and IPSG - in charge of stabilometry and electromyography; EECA, CMH, NCM, TCS - responsible for the execution of the treatments; FP, YE in charge of the data processing and analysis; CFA - responsible for equipment used in the study; MBSG and BRBB - in charge of patients recruitment and performed the reviews of the manuscript; APA - translated the manuscript to English; DABG -s in charge of the laboratory and coordination of the study. All authors read and approved the final manuscript.
